# Selective ultrasound screening is inadequate to identify patients who present with symptomatic adult acetabular dysplasia

**DOI:** 10.1007/s11832-014-0620-1

**Published:** 2014-11-06

**Authors:** Ernest L. Sink, Benjamin F. Ricciardi, Katrina Dela Torre, Charles T. Price

**Affiliations:** 1 Center for Hip Pain and Preservation, Hospital for Special Surgery, 541 East 71th Street, 10021 New York, NY USA; 2 Arnold Palmer Hospital for Children, Orlando, FL USA

**Keywords:** Developmental dysplasia of the hip, Selective ultrasound screening of newborn hip dysplasia, Pelvic osteotomy, Hip dysplasia

## Abstract

**Purpose:**

One goal of neonatal screening for developmental dysplasia of the hip (DDH) is the prevention of late surgery. However, the majority of patients with acetabular dysplasia at skeletal maturity are not diagnosed with DDH during infancy. Selective ultrasound screening may identify patients with neonatal hip instability, but may be ineffective for the prevention of dysplasia presenting in adulthood. The purpose of this study is to identify the prevalence of risk factors for DDH that would have warranted selective ultrasound screening in patients with symptomatic acetabular dysplasia after skeletal maturity.

**Methods:**

A prospective hip specialty center registry was used to identify 68 consecutive skeletally mature patients undergoing corrective osteotomy for symptomatic acetabular dysplasia. Risk factors for DDH evaluated in all patients included sex, family history of hip osteoarthritis or DDH, breech, method of delivery, previous hip treatments, and birth order. Radiographs [lateral center edge angle (CEA), anterior CEA, Tönnis grade, and Tönnis angle] were measured preoperatively.

**Results:**

Sixty-seven females and one male were identified. No patients were previously diagnosed with DDH or received treatment for their hips. The majority of patients (85.3 %) did not meet selective ultrasound screening guidelines following a stable neonatal hip exam and, therefore, would not have been screened in a selective screening program. Of the findings outside of screening guidelines, 98.5 % were females, 52.9 % were first born, and 36.8 % had a family history of hip osteoarthritis.

**Conclusions:**

The majority (85.3 %) of patients with symptomatic acetabular dysplasia at skeletal maturity would not have met current recommendations for selective ultrasound screening in the USA had they been born today.

## Introduction

Acetabular dysplasia is a common cause of hip osteoarthritis and the most common cause of hip arthroplasty in women younger than 50 years of age. [1, 2]. Hip instability and acetabular dysplasia affects up to 4 % of newborn infants [3–6]. Untreated or undetected developmental dysplasia of the hip (DDH) may result in abnormal acetabular development during infancy, and this may lead to an increased risk of symptomatic acetabular dysplasia at later ages [7, 8]. Neonatal hip screening protocols, including both physical examination and/or imaging studies, have been implemented in many countries to promote early diagnosis of DDH, allowing for less invasive treatments, and possibly reducing the rate of late open surgery [5–11]. Some centers recommend universal screening to further decrease the burden of hip dysplasia in later life [7, 8].

Serial clinical examination is widely accepted for all newborns to improve early detection of DDH; however, recommendations for ultrasound screening have considerable regional variation [5, 6, 9]. Selective ultrasound screening in high-risk newborns following a normal neonatal examination has been recommended in the USA by organizations such as the American Academy of Pediatrics (AAP) as an alternative to universal ultrasound screening [9, 12, 13]. Selective secondary ultrasound screening is specifically recommended for infants born in the breech position or with a positive family history for DDH. The reports of selective screening are mixed: some studies indicate a decrease in late dysplasia surgery, while others conclude no effect on the incidence of late surgery compared to physical exam alone [6, 12, 14, 15]. Additionally, some series have concluded that a large percentage of children with the need for late open surgery would be missed by current selective screening recommendations [10, 15, 16].

The majority of current studies have focused on the impact of selective ultrasound screening on late surgery in skeletally immature patients. The effect of selective ultrasound screening on operative intervention for dysplasia identified at skeletal maturity has been less frequently studied [3, 5, 7, 8, 14, 15, 17–20]. The purpose of this study is to identify the prevalence of risk factors for neonatal DDH that would have warranted selective ultrasound screening in a consecutive series of skeletally mature patients with symptomatic acetabular dysplasia.

## Materials and methods

Patients were enrolled in this study through our prospective single-center hip preservation registry, which was established at our institution in March 2010. We serve as a tertiary referral center for hip disorders and hip preservation surgery. All patients undergoing surgery, including hip arthroscopy, periacetabular osteotomy (PAO), femoral osteotomy, and surgical hip dislocation (SHD), are enrolled prospectively from four surgeons performing procedures at our institution. Patients with symptomatic acetabular dysplasia presenting between June 2011 and December 2013 were eligible for this study. Radiographic criteria for acetabular dysplasia utilized in this cohort were a lateral center edge angle (CEA) on the anteroposterior radiograph ≤20° and/or an anterior CEA ≤20° on the false-profile radiograph. Exclusion criteria included patients with neuromuscular dysplasia, teratologic hip dislocation, or inability to confirm birth history.

Based on our inclusion criteria, we retrospectively identified a consecutive series of 68 skeletally mature patients recommended to have corrective osteotomy for symptomatic hip dysplasia. The surgical decision was based on a careful history, exam, plain radiographs, magnetic resonance imaging (MRI), and computed tomography (CT) scan to confirm hip dysplasia as the etiology of hip symptoms. Prospective data were collected at the time of initial consultation from all patients including a questionnaire regarding their birth history and risk factors for DDH including: family history of hip pain in first- or second-degree relatives, hip surgery, or DDH; whether they were breech, method of delivery, previous treatment or surgery on their hips, and birth order. A follow-up call was performed to confirm this documented history prior to the study.

Radiographic studies (radiographs, MRI, and CT) were analyzed for each patient at the time of their initial consultation. Patients with a lateral CEA, anterior CEA, Tönnis grade, and Tönnis angle were recorded by the interviewing surgeon (ELS) from plain radiographs in a standard fashion.

Continuous variables are presented as the mean ± standard deviation or mean (range), as indicated. Categorical variables are presented as the percentage of the total cohort as having the described features (SAS version 9.3, SAS Institute, Cary, NC).

## Results

There were 68 patients included in the study (Table 1). The average age was 26.4 years (range 13–47 years). There were 67 (98.5 %) females and 1 (1.5 %) male. The left hip presented as the symptomatic hip in 21 (30.9 %) cases, and the right hip in 47 (69.1 %) cases. The duration of pain was 6 months to 288 months (median 24 months). The mean lateral CEA was 14.7 ± 6.2° and the mean anterior CEA was 14.5 ± 9.3° (Table 2). The Tönnis grade was zero in 48.5 %, one in 41.2 %, and two in 10.3 %. The mean Tönnis angle was 16.8 ± 6.5°.

**Table 1:** Demographic characteristics of patients presenting with symptomatic hip dysplasia after skeletal maturity

Demographic parameters, *N* = 68 patients	
Age (years)	26.4 (range 13–47)
Sex (% female)	98.5
Laterality (% right)	69.1
Symptom duration (months)	24 (range 6–288)

**Table 2:** Radiographic parameters of the symptomatic hip in patients presenting at skeletal maturity with symptomatic hip dysplasia

Radiographic parameters, *N* = 68 patients	
Lateral CEA (°)	14.7 (6.2)
Anterior CEA (°)	14.5 (9.3)
Tönnis angle (°)	16.8 (6.5)
Tönnis grade	
0	33 (48.5%)
1	28 (41.2%)
2	7 (10.3%)

All continuous variables are presented as mean ± standard deviation. The Tönnis grade is presented as the number (percentage) of total patients with morphologic features

The questionnaire results about risk factors for hip disease are shown in Table 3. Eight patients (11.8 %) were confirmed breech. A family history of DDH was present in two additional patients (2.9 %). Therefore, current guidelines would recommend selective ultrasound screening in 10/68 patients (14.7 %) of this cohort.

**Table 3:** Risk factors for developmental dysplasia of the hip (DDH) in patients presenting with symptomatic hip dysplasia after skeletal maturity

Risk factor	Patients with positive history (%), *N* = 68 patients
Breech	11.8
Family history of DDH	2.9
Family history of hip osteoarthritis	36.8
Prematurity	7.4
First born	52.9
Previous childhood treatment for hip disorder	0

Characteristics that were identified but are not current indications for selective ultrasound screening included 36 patients (52.9 %) that were first born, 25 patients (36.8 %) with a family history of hip osteoarthritis in a first- or second-degree relative (but not confirmed by the family as DDH), and 5 patients (7.4 %) who were born premature. Despite the presence of symptomatic hip dysplasia at skeletal maturity, no patients in this series were diagnosed with DDH as children or received any treatment for their hips.

## Discussion

Skeletally mature patients with symptomatic acetabular dysplasia, despite current protocols for early identification and treatment of DDH, may result from inadequate infantile screening methods, inadequate screening protocols, or treatment failure. It is also possible that adult acetabular dysplasia represents a different disease process than infantile DDH, with a later onset of development outside of the current screening period [21, 22]. In this consecutive series of skeletally mature hips with symptomatic acetabular dysplasia, only 10/68 patients (14.7 %) had risk factors for DDH at birth that would be evaluated by current selective ultrasound screening recommendations in neonates with a stable hip exam. Therefore, 85.3 % of the patients in this cohort would have been undetected by current selective ultrasound screening recommendations.

The overall goal of screening is to decrease the incidence of late acetabular dysplasia and hip instability that requires surgical treatment. Early diagnosis of DDH in the newborn period allows less invasive treatment such as Pavlik harness, and this may decrease the risk of future invasive surgery for acetabular dysplasia [8, 11, 19, 20, 23]. Early detection and intervention of neonatal DDH may decrease the risk for development of acetabular dysplasia in skeletally mature patients. The role of selective ultrasound screening in patients with risk factors remains controversial. In 2006, the US Preventive Services Task Force concluded, based on its systematic review, that there was not enough evidence to recommend routine screening in the neonatal period (clinical or ultrasound) [11]. The Canadian task force reported that routine clinical exam in the newborn period for hip instability decreased late operative rates; however, the addition of selective ultrasound screening did not result in further reductions [6]. Recently, Sanghrajka et al. [10] reported a series of patients treated with late surgery at their center in England and concluded that only a minority of patients (23 %) would have been identified using selective ultrasound screening based on risk factors. Cost-effectiveness is critical to any screening protocols. A decision analysis study by Mahan et al. [13] suggested that selective ultrasound screening (breech, family history of DDH) with universal physical examination screening may be the most cost-effective strategy in the USA, unless the rate of missed dysplasia was greater than 4/1,000.

Current screening recommendations may have little impact on changing the incidence of symptomatic acetabular dysplasia after skeletal maturity. Breech position and family history of DDH are the two major risk factors included in recommendations for selective screening in the USA [9, 11, 24–26]. In our study, patients with acetabular dysplasia at skeletal maturity rarely reported a positive family history of DDH (2/68 patients). In contrast, a family history of hip osteoarthritis in any first- or second-degree relative was common in this population (25/68). This is consistent with the findings of Schiffern et al. [27] and Lee et al. [21], which suggest an increased association of hip osteoarthritis amongst first- and second-degree relatives of patients with acetabular dysplasia. Expanding the definition from a family history of DDH to any family history of hip pain and osteoarthritis in first- and second-degree relatives may increase the percentage of neonatal diagnoses with selective screening. Another major risk factor in DDH is female sex [11, 24]. Our cohort of patients with adult dysplasia requiring treatment was almost exclusively female. Expanding current selective ultrasound screening protocols to include all females, or at least all first-born females, may increase early identification of these patients. Male patients are less likely to need treatment after selective or general ultrasound screening; thus, expanding screening in males beyond current recommendations appears unnecessary [28, 29].

Another possible explanation for acetabular dysplasia after skeletal maturity is ineffective diagnostic measures. Current protocols of serial clinical exams and ultrasound screening for DDH are primarily focused on detecting neonatal hip instability, but instability resolves spontaneously in many cases. These approaches may not address cases involving stable acetabular dysplasia without evidence of hip instability. New approaches to diagnosis and treatment may be needed to detect more subtle cases of stable hip acetabular dysplasia in infancy. Acetabular dysplasia is common in Scandinavian countries, and routine radiographs of the pelvis at 4–5 years of age have been suggested in order to treat dysplasia before the onset of arthritis [30]. Whether early treatment would benefit patients with more subtle stable acetabular dysplasia is an unresolved question; however, there is evidence in canines and in humans that proper positioning of the hips during the neonatal period may benefit acetabular development [31].

Acetabular dysplasia after skeletal maturity may have developed post-infancy and represents a distinct disease entity from infantile DDH that may not be affected by screening more of the population in infancy [21, 22]. Lee et al. [21] found that male sex, bilateral hip pathology, and family history of early total hip arthroplasty (<65 years of age) was more common in adult- or adolescent-onset acetabular dysplasia relative to a population of patients with infantile DDH, who tended to have a family history of DDH and were predominantly female. The authors suggested that infantile DDH and adult- or adolescent-onset DDH may represent separate disease entities; however, inadequate selective screening criteria could still explain the low rates of a family history of DDH seen in the adult dysplasia group [21]. Additionally, our data does not support the male predominance in adult acetabular dysplasia found by Lee et al., suggesting differences in our patient populations or referral patterns. Universal ultrasound screening has been reported in Austria to reduce the incidence of late surgery for all ages, including patients aged 15–35 years, which suggests that expanded ultrasound screening and higher treatment rates in neonates may have an impact on the incidence of skeletally mature dysplasia [18]. Our study suggests that selective ultrasound screening may be inadequate to identify delayed development of dysplasia outside of the neonatal period. Despite these findings, further studies examining the origins of infantile DDH and adolescent/adult hip dysplasia are needed in order to determine the contribution of missed screening versus post-infancy development of adult hip dysplasia.

There are several limitations to this study. Patients in this study presented to a tertiary referral center with hip pain and concomitant acetabular dysplasia. The diagnosis was based on a detailed physical exam, radiographs (anteroposterior and false-profile radiographs), MRI of the hip, and 3D CT scan. There are limits to a broad population-based conclusion with this study, since these were only symptomatic patients that presented to the authors’ center. It is unclear whether asymptomatic patients with adult dysplasia would have different characteristics to this population. Even if the patients in this cohort were theoretically screened, they may still present with symptoms attributed to dysplasia due to a failure of the screening mechanism, such as misinterpreted clinical examination or ultrasound findings, or, as previously mentioned, a lack of acetabular development that may not be recognizable in the infant hip.

In summary, only 14.7 % of skeletally mature patients presenting with symptomatic acetabular dysplasia would meet current criteria for selective ultrasound screening for a stable hip. Current screening may improve the incidence of early diagnosis and treatment of hip dysplasia in the high-risk group (breech and family history of DDH), but it may not have a significant impact on the incidence of skeletally mature acetabular dysplasia. Expanding selective ultrasound screening criteria to include any family history of hip osteoarthritis and/or female sex, especially first-born females, may further identify a significant group of patients who have risk factors for developing acetabular dysplasia at skeletal maturity. Additionally, the role of expanding screening modalities to older age groups in patients at increased risk for adult dysplasia should continue to be investigated in the future. It remains unclear whether increased identification and treatment of patients with infantile DDH would decrease the rates of symptomatic acetabular dysplasia in skeletally mature adults. Further studies are needed in order to fully define appropriate screening protocols.
